# Direct regulation of the natural competence regulator gene *tfoX* by cyclic AMP (cAMP) and cAMP receptor protein (CRP) in *Vibrios*

**DOI:** 10.1038/srep14921

**Published:** 2015-10-07

**Authors:** Rui Wu, Meng Zhao, Jing Li, He Gao, Biao Kan, Weili Liang

**Affiliations:** 1 National Institute for Communicable Disease Control and Prevention, and State Key Laboratory of Infectious Disease Prevention and Control, Chinese Centre for Disease Control and Prevention, Changping, Beijing, People’s Republic of China; 2 Collaborative Innovation Centre for Diagnosis and Treatment of Infectious Diseases, Hangzhou, People’s Republic of China

## Abstract

TfoX (Sxy) and CRP are two important competence activators. The link between *tfoX* and CRP has been shown in *H. influenza* but lacking evidence of direct interaction. Recently a Sxy-dependent CRP (CRP-S) site autoregulating Sxy was reported in *E. coli*. Here, we show that the cAMP-CRP complex transcriptionally regulates *tfoX* expression through multiple canonical CRP (CRP-N) sites in *Vibrios.* This conclusion is supported by an analysis of the *tfoX* mRNA levels and *tfoX* transcriptional reporter fusions. The reduced expression of *tfoX*^*VC*^ was restored by trans-complementation of *crp* in ∆*crp* and by exogenous cAMP in ∆*cya*. A promoter deletion analysis and the site-directed mutagenesis of the putative CRP-N sites revealed the presence of two functional CRP-N sites. The direct binding of cAMP-CRP to the *tfoX*^*VC*^promoter was demonstrated by EMSA assays. Additionally, the transcriptional start site (TSS) of *tfoX*^*VF*^ in *V. fluvialis* was determined, and −10/−35 regions were predicted. Further comparison of the *tfoX* promoter in *Vibrios* revealed the existence of similar −10 motifs and putative CRP-N sites, indicating the conserved mechanism of CRP regulation on *tfoX*. Our study demonstrates the direct binding of the cAMP-CRP complex to *tfoX* promoter, and broadens the understanding of the molecular mechanism regulating *tfoX* in *Vibrios*.

Many bacterial species can take up environmental DNA under natural conditions, most of which is degraded in the cytoplasm for reuse. However, the DNA that escapes degradation might be incorporated into the host chromosomes by RecA-dependent homologous recombination^1^,^2^,^3^,^4^,^5^,^6^. This is a very complicated phenomenon with regard to its biological function, machinery components and the regulated and regulatory genes involved. In general, the process requires certain cellular structures, like type IV pili or related pseudopili, and many competence-related proteins, and interferes with other regulation systems, such as carbon catabolite repression (CCR), quorum sensing and nucleotide scavenging systems^7^,^8^,^9^. Although many advances have been made in this field, a clear picture of the molecular details of natural competence remains to be revealed.

Most of our understanding of the genetics and molecular mechanisms of competence originally came from studies of *H. influenzae*^3^,^10^. Nutrient starvation and the depletion of nucleotide pools induce natural competence/transformation in these bacteria^3^,^8^. The competence regulon of *H. influenzae* is characterized by a promoter-associated 22 bp competence regulatory element (CRE, 5′-T_4_G_5_**C**_**6**_G_7_A_8_–(N6)–T_15_C_16_**G**_**17**_C_18_A_19_-3′) that is closely related to the cAMP receptor protein (CRP) binding consensus (5′-T_4_G_5_**T**_**6**_G_7_A_8_–(N6)–T_15_C_16_**A**_**17**_C_18_A_19_-3′)^10^. The sequence has C and G instead of T and A at the highly conserved symmetrical positions 6 and 17, which are positions where CRP bends DNA^11^. The presence of cAMP and Sxy allows CRP to bind at CRE sites to activate the transcription of competence genes, and the affinity of these sites for CRP binding was experimentally demonstrated^8^,^10^,^12^. The terms CRP-S and CRP-N were respectively introduced to designate the new Sxy-dependent and canonical (Sxy-independent) CRP sites^12^.

In contrast to the naturally competent *H. influenzae*, *Vibrio cholerae*, the causative agent of cholera epidemics, was not known to be competent until Meibom *et al.* published their study in 2005 5. That study first demonstrated that chitin, a polymer of β-1,4-linked *N*-acetylglucosamine (GlcNAc), induces natural competence in *V. cholerae*^5^. In *V. cholerae*, competence development is triggered by a chitin-induced transcription regulator, Tfox^*VC*^, an ortholog of Sxy in *H. influenzae*, and a quorum-sensing regulator, HapR^5^. *V. cholerae* have counterparts of most *H. influenzae* Sxy-dependent, CRP-regulated genes^12^, and its competence phenotype is subject to catabolite repression^5^, which is positively regulated by two environmental signals, nutritional stress^5^ and nucleoside scavenging, similar to *H. influenzae*^7^. A natural competence phenotype was subsequently demonstrated to be a shared trait in other *Vibrio* species, such as *V. fischeri*^13^, *V. vulnificus*^14^,^15^, and *V. parahaemolyticus*^16^.

Sxy (TfoX) and CRP are two major shared activators that control the development of competence in the families *Pasteurellaceae*, *Enterobacteriaceae*, and *Vibrionaceae*^12^. Both *sxy* (*tfoX*) or *crp/cya* knockout mutants are unable to become competent^17^,^18^,^19^. Due to its vital role in regulating natural competence, the regulatory mechanism involving *sxy* (*tfoX*) has attracted increasing attentions from researchers. The secondary structure of *sxy* mRNA greatly increases its translational efficiency and promotes competence development in *H. influenzae*^20^. Besides chitin, a polymer of β-1,4-linked N-acetylglucosamine (GlcNAc), chitin disaccharide (GlcNAc)_2_ was also shown to be a minimum inducer of *tfoX* expression at both the transcriptional and translational levels in *V. cholerae*, and the corresponding *cis*-acting elements were previously identified^21^. A small regulator RNA, *tfoR*, activates the translation of *tfoX* in response to the presence of (GlcNAc)_2_ or chitin^22^.

CRP is one of major players in carbon catabolite repression (CCR)^19^, which allows bacteria to quickly respond to environmental changes by repressing genes responsible for the uptake, metabolism and assimilation of less favorable carbon sources when rapidly metabolizable carbohydrates, such as glucose, are present^19^,^23^,^24^. It has been shown that CRP plays a crucial role in the *V. cholerae* life cycle by regulating the expression of virulence factors (i.e., cholera toxin and toxin co-regulated pilus)^25^, affecting exopolysaccharide biosynthesis and rugose colonial morphology^26^, quorum sensing, motility, and multiple genes required for the survival of *V. cholerae* in the human host and the environment^27^,^28^.

The necessity of CRP and cAMP for chitin-induced competence was proven based on their interactions in the three interlinked aspects: they are required for the efficient colonization of the chitin surface, contribute to chitin degradation and utilization, and increase the expression of competence genes^19^. Our previous global gene expression profile based on a microarray analysis revealed that *tfoX* was down-regulated in a ∆*crp* mutant (Supplemental data)^27^, but this was not confirmed. In the present study, we add new information about the positive regulation of CRP on the development of competence by demonstrating the positive transcriptional regulation and direct binding of the cAMP-CRP complex on the *tfoX* promoter region in both *V. cholerae* and *V. fluvialis*, the latter of which is an emerging foodborne pathogen implicated in outbreaks and sporadic cases of cholera-like bloody diarrhea, and causes increasing public health concerns^29^,^30^. Many aspects of the ecology, epidemiology and pathogenesis of *V. fluvialis* remain to be investigated.

## Results

### Effect of CRP on *tfoX* gene expression

Yamamoto’s works greatly improved the knowledge of the regulation of *tfoX*^*VC*^ in *V. cholerae* by identifying chitin disaccharide (GlcNAc)2 as the minimum inducer of chitin-dependent competence, by mapping the TSS, and the transcriptional and translational *cis*-acting elements, and by distinguishing the sRNA *tfoR* regulation^21^,^22^. Using this knowledge, combined with the prior clues provided by microarray data^27^, we reexamined the promoter region of *tfoX*^*VC*^ and found a potential CRP-N binding site centered at −84.5 relative to the TSS, which contained a perfect TGTGA half-site and a 4/5 matched TCTCA half-site for the DNA binding domains of the active CRP dimer. These findings strongly suggested the possibility that CRP regulates *tfoX*^*VC*^ expression.

**Figure 1:**
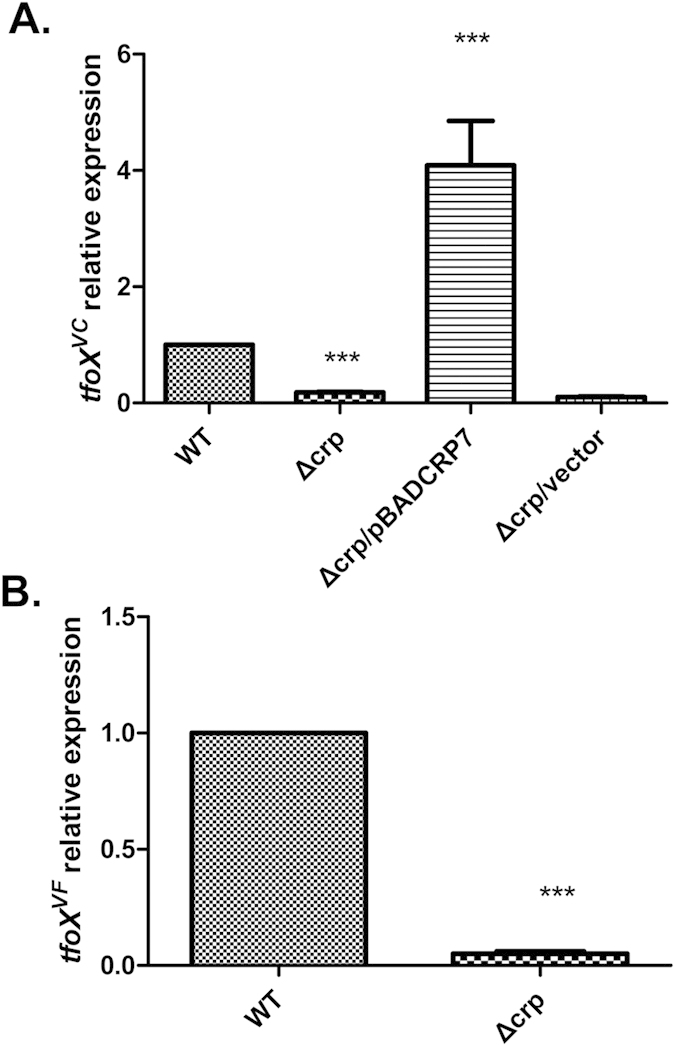
Effects of CRP on the *tfoX* gene expression. (**A**) *V. cholerae* strains C7258∆lacZ (WT), WL7258∆lacZ (∆*crp*), and WL7258∆lacZ containing the wild-type *crp* gene in trans and the control vector were grown in LB medium to late-log phase. (**B**) *V. fluvialis* strains 85003 (WT) and 85003∆*crp* (∆*crp*) were grown in LB medium, and cells were collected at the late-log phase. The *tfoX*^*VC*^ and *tfoX*^*VF*^ mRNA abundances were measured by qRT-PCR. The “WT” bar was set to 1 and used as a reference to calculate subsequent expression values. Error bars indicate the standard deviations of three independent cultures. ***Significantly different from the wild-type strain (*t*-test, P < 0.05).

To determine whether CRP was involved in the expression of *tfoX*^*VC*^, we examined the *tfoX*^*VC*^ mRNA level in wild type and isogenic mutant *crp* via qRT-PCR (Fig. 1A). Deletion of the *crp* gene resulted in much lower expression of *tfoX*^*VC*^, which confirmed our microarray data. For further confirmation, we performed a complementation test by introducing plasmid pBADCRP7, which expresses biologically active CRP protein from the *araBAD* promoter. As shown in Fig. 1A, the *tfoX*^*VC*^ expression was restored to a much higher level than the WT level in the pBADCRP7-complemented *crp* mutant. As expected, inclusion of the control vector did not restore the *tfoX*^*VC*^ expression.

To verify and expand the above finding that CRP was required for the expression of *tfoX*, we further examined the *tfoX* mRNA level in an emerging foodborne pathogen, *V. fluvialis*, and its isogenic *crp* mutant. The *crp* mutant was constructed by allelic exchange as described in the Materials and Methods section. Compared to the wild type pathogen, the *tfoX*^*VF*^ mRNA level in the corresponding *crp* deletion mutant was significantly reduced (Fig. 1B). Taken together, these results indicate that the requirement of CRP for the expression of *tfoX* is a common feature in *Vibrios*. However, it was unclear whether this requirement is dependent on a direct interaction, or whether it may be a pleiotropic (or secondary) effect, because CRP is a global regulator and affects numerous genes.

### Effects of *cya* mutation on the *tfoX* gene expression

**Figure 2:**
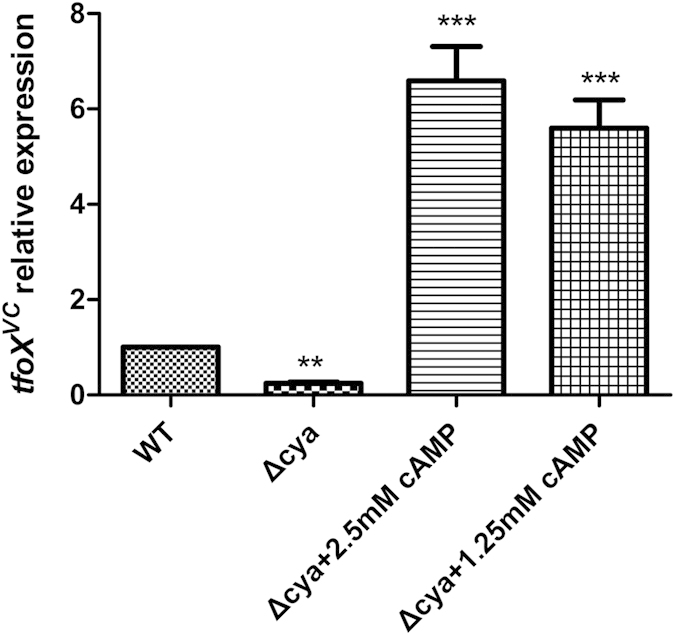
Effects of *cya* mutation on the *tfoX* gene expression. Overnight cultures of WL7259 (∆*cya*) were diluted 100-fold in LB and grown to OD600 0.5. Each culture was divided into three portions, and exogenous cAMP (Sigma Chemical Co.) was added to a final concentration of zero (control), 1.25, or 2.5 mM. The cultures were incubated at 37 °C for 1 h and the *tfoX*^*VC*^ mRNA abundance was measured by qRT-PCR. The “WT” bar was set to 1 and used as a reference to calculate subsequent expression values. Error bars indicate the standard deviations of three independent cultures. **Significantly different from the wild-type strain (*t*-test, P < 0.05). ***Significantly different from the wild-type strain (*t*-test, P < 0.001).

To determine if the *tfoX*^*VC*^ dependency on CRP can be fully accounted for by the cAMP binding to CRP, we measured the *tfoX*^*VC*^ expression in WL7259 and WL7259 supplemented with 1.5 or 2.5 mM cAMP in the culture medium. As shown in Fig. 2, there was significantly lower expression of *tfoX*^*VC*^ in the WL7259 lacking adenylate cyclase compared to the wildtype strain. Furthermore, the expression of *tfoX*^*VC*^ was fully restored by supplementing the medium with exogenous cAMP. The activity of CRP is determined by the intracellular concentration of its allosteric activator, cAMP^31^. Consistent with this, the *tfoX*^*VC*^ level in the strain with extra cAMP supplementation was more than 10-fold higher than that of the wild type strain. These results suggest that the expression of *tfoX*^*VC*^ is under positive regulation by the cAMP-CRP complex. However, it was still unclear whether the dependence on both CRP and cAMP indicates the presence of a direct effect, and further evidence was required to clarify this point.

### CRP activates the transcription of *tfoX*

**Figure 3:**
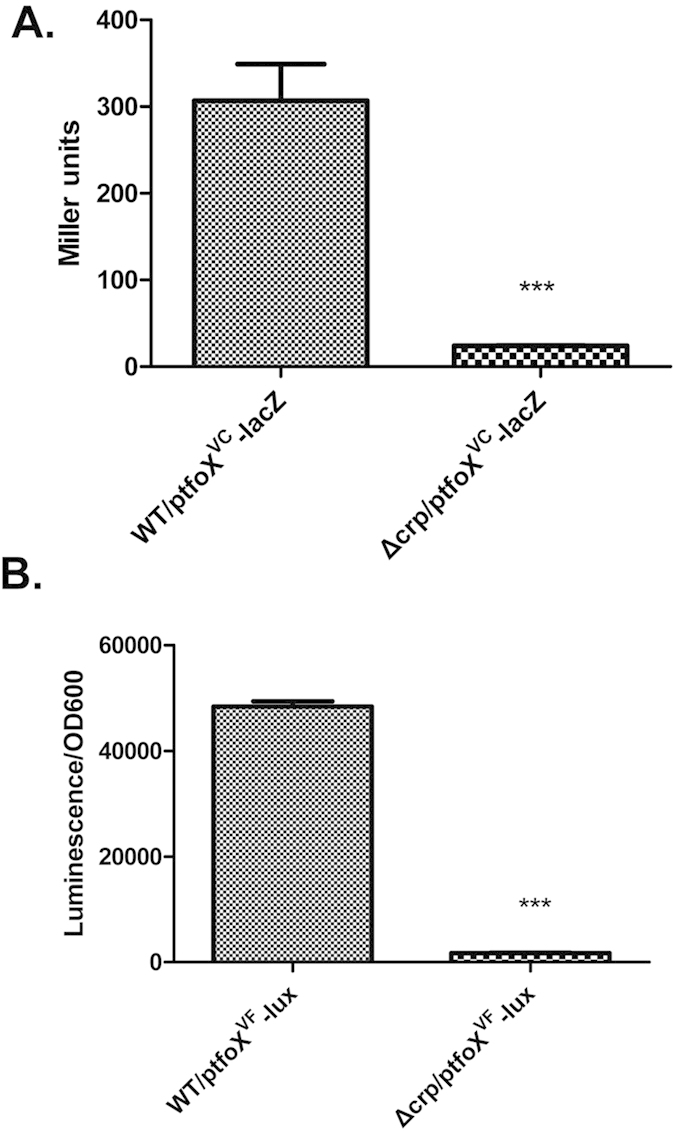
Transcriptional fusion analysis of the cAMP–CRP regulation of *tfoX* expression. (**A**) The *β*-galactosidase expression in *V. cholerae* strains C7258∆lacZ (WT) and WL7258∆lacZ (∆*crp*) containing a p*tfoX*^*VC*^–*lacZ* transcriptional fusion. (**B**) The luminescence activity in *V. fluvialis* strains 85003 (WT) and 85003∆*crp* (∆*crp*) containing a p*tfoX*^*VF*^–*lux* transcriptional fusion. All strains were grown at 37 °C with shaking to the mid-log phase. The *β*-Galactosidase and bioluminescence activity were measured as described in the Methods. Error bars indicate the standard deviations of three independent cultures. ***Significantly different from the wild-type strain (*t*-test, P < 0.005).

To determine if the CRP-mediated regulation of *tfoX* occurs at the level of transcription, we constructed transcriptional reporter plasmids by fusing the promoter regions of *tfoX*^*VC*^ and *tfoX*^*VF*^to the reporter genes *lacZ* and *luxCDABE*, respectively. The −10 and −35 motifs of *tfoX*^*VC*^ promoter have been determined in Yamamoto’s work^21^. The promoter of *tfoX*^*VF*^ was predicted with sequence analogy of the *tfoX*^*VC*^ promoter and TTS determined in our study (below part). In order to retain the full promoter activity, the sequences extending 408 bp and 381 bp upstream of *tfoX*^*VC*^ and *tfoX*^*VF*^ translational start sites were respectively amplified as promoter regions. The reporter constructs were transferred into the corresponding wild-type strains and the isogenic *crp* mutants. Significant differences in *β*-galactosidase expression (Fig. 3A) and luminescence activity (Fig. 3B) were detected between the WT and ∆*crp* containing the reporter constructs on both the *V. cholerae* and *V. fluvialis* backgrounds. Combined with the results about the *tfoX* mRNA expression level, these results strongly demonstrated that the promoter activity of *tfoX* is dependent on CRP in *Vibrio* species, i.e. CRP is directly required for *tfoX* expression.

### Deletion analysis of the *tfoX* ^*VC*^ promoter region

**Figure 4:**
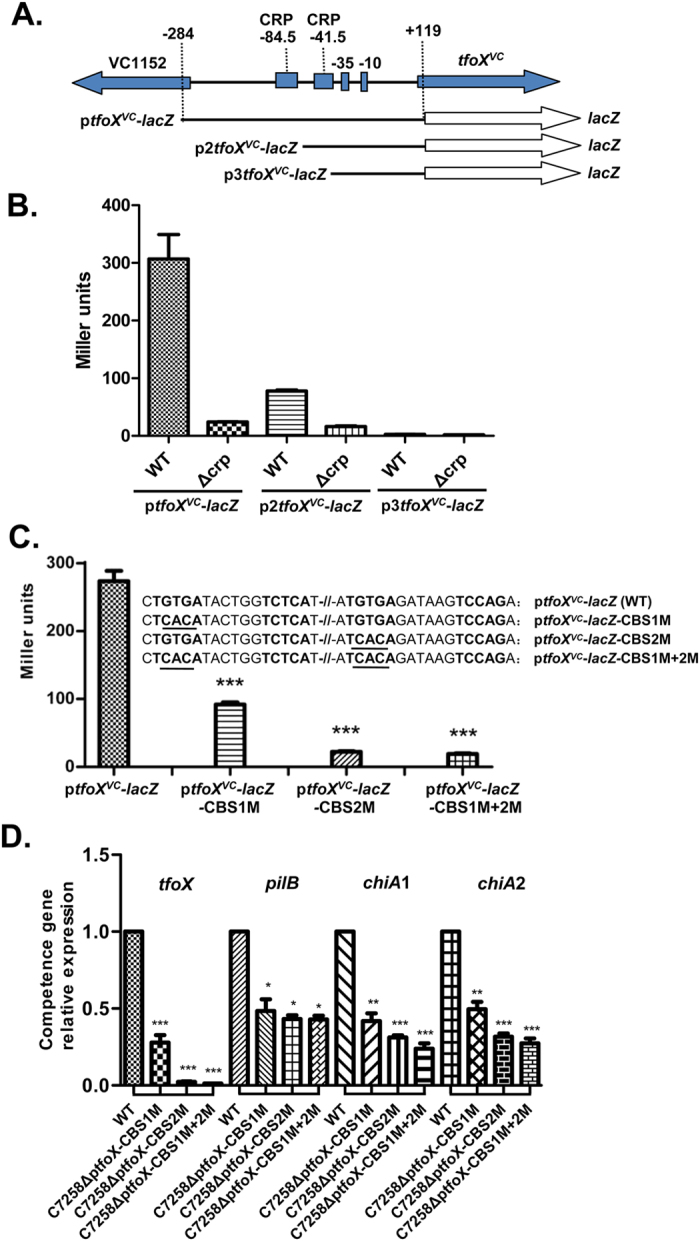
Effects of putative CRP binding sites on the *tfoX*^*VC*^ expression. (**A**) The structural organizations of the *tfoX*^*VC*^ promoter and transcriptional fusions. Two putative CRP binding sites were found in the upstream region of *tfoX*^*VC*^ centered at positions −84.5 and −41.5. (**B**) A deletion analysis of the *tfoX*^*VC*^ promoter. *V. cholerae* strains C7258∆lacZ (WT) and WL7258∆lacZ (∆*crp*) containing the p2*tfoXVC*–*lacZ* and p3*tfoXVC*–*lacZ* fusion, respectively, were grown at 37 °C to mid-log phase. (**C**) The promoter activities of wild-type and CBS mutated fusions. C7258∆lacZ containing p*tfoXVC*–*lacZ*, p*tfoXVC-lacZ-*CBS1M, p*tfoXVC-lacZ-*CBS2M, or p*tfoXVC-lacZ-*CBS1M+2M were grown at 37 °C to the mid-log phase. The *β*-galactosidase activity was measured as described in the Methods. The mutated bases in fusions were constructed by site-directed mutagenesis and underlined. (**D**) *V. cholerae* strains C7258 (WT), C7258∆p*tfoX*-CBS1M, C7258∆p*tfoX*-CBS2M and C7258∆p*tfoX*-CBS1M+2M were grown in LB medium to late-log phase. The *tfoX*^*VC*^ and *pilB*, *chiA*-1 and *chiA*-2 mRNA abundances were measured by qRT-PCR. Error bars indicate the standard deviations of three independent cultures. The “WT” bar was set to 1 and used as a reference to calculate subsequent expression values. ***Significantly different from the wild-type strain (*t*-test, P < 0.0003). *Significantly different from the wild-type strain (*t*-test, P < 0.05).

To further delineate the *cis* DNA sequences in the *tfoX* promoter region required for CRP activation, we constructed additional transcriptional fusions containing 5′ deletions of the *tfoX*^*VC*^ promoter (Fig. 4A). Plasmid p_2_*tfoX*^*VC*^-*lacZ* contained a DNA sequence extending from −74 bp upstream of the TSS, lacking the previously predicted CRP-binding sequence (TGTGA-N6-TCTCA); while p_3_*tfoX*^*VC*^-*lacZ* contained an even shorter sequence starting from the putative −35 element. As shown in Fig. 4B, p_3_*tfoX*^*VC*^-*lacZ* exhibited the lowest *β*-galactosidase activity, and no difference was observed between the WT and ∆*crp*. Surprisingly, plasmid p_2_*tfoX*^*VC*^-*lacZ* retained substantially high promoter activity, and a significant difference in *β*-galactosidase activity was still retained for the WT and ∆*crp*, indicating that the encompassed promoter region contains DNA sequences necessary for CRP activation.

Further inspection of the promoter region revealed another suboptimal CRP binding site (**TGTGA**GATAAG**TCCAG**), with three mismatches from the consensus motif on the one half-site (TCACA). This binding site is centered at −41.5, encompasses the predicted −35 hexamer, and thus extends into the region generally bound by the RNA polymerase (RNAP). Based on these results, we inferred that the *tfoX*^*VC*^promoter resembles or belongs to the Class III CRP-dependent promoter, which requires multiple activator molecules for full transcriptional activation. In this case, it requires two CRP molecules. Specifically, one binding site is centered at position −84.5 as in the class I CRP-dependent promoters; the other is centered at −41.5, exactly like the class II CRP-dependent *galP1* promoter^32^. The tandem binding of CRP causes synergistic effects on transcriptional activation, with the best effects resulting from the binding to a site situated 40 or 50 bp upstream^33^. The 43-bp spacer of the two binding sites (center-to-center distance) of *tfoX*^*VC*^seems to be an optimal distance that produces the best synergistic effects.

### Site-directed mutagenesis of the CRP-binding site at the promoter of *tfoX* ^*VC*^

The p*tfoX*^*VC*^-*lacZ* reporter fusion contains the full length functional *tfoX* promoter region so that it encompasses two putative CRP-binding sites which were designated as CBS1 and CBS2, respectively. To further verify and distinguish the roles of each CRP-binding site in *tfoX*^*VC*^ transcription, we substituted the consensus GTG for ACA in the half-site of CBS1, CBS2, and both by site-directed mutagenesis (Fig. 4C). The promoter activities of the resultant mutant fusions p*tfoX*^*VC*^-*lacZ*-CBS1M, p*tfoX*^*VC*^-*lacZ*-CBS2M and p*tfoX*^*VC*^-*lacZ*-CBS1M+2M, were determined. As shown in Fig. 4C, the mutation of CBS1 resulted in a major decrease in the *β*-galactosidase activity of the p*tfoX*^*VC*^-*lacZ*-CBS1M fusion compared to the wild-type p*tfoX*^*VC*^-*lacZ*, which is consistent with the results of the p_2_*tfoX*^*VC*^-*lacZ* construct. As expected, p*tfoX*^*VC*^-*lacZ*-CBS1M+2M showed the lowest *β*-galactosidase activity due to the mutations of both CBS1 and CBS2 in the promoter region. Unexpectedly, mutation of CBS2 also results in a very low level of *β*-galactosidase activity in p*tfoX*^*VC*^-*lacZ*-CBS2M, similar to that observed for the double mutant fusion p*tfoX*^*VC*^-*lacZ*-CBS1M+2M. This result indicates that the CBS1 centered at −84.5 cannot efficiently initiate the transcription of the *tfoX*^*VC*^promoter alone, and CBS2 is required for the synergistic activation of the *tfoX*^*VC*^promoter by CBS1.

To further determine the mutation of either CBS or both in the *tfoX* promoter has an effect in competence development or on natural transformation *in vivo*, We subsequently introduced the site specific mutations to CBS1, CBS2 and both on the *V. cholerae* chromosomal *tfoX* promoter, and measured the mRNA levels of *tfoX*, and *tfoX*-induced genes, *pilB*, *chiA*1 and *chiA*2, which are all required for competence in *V. cholerae*^5^. As shown in Fig. 4D, the mRNA levels of *tfoX*, *pilB*, *chiA*1 and *chiA*2 in C7258∆p*tfoX*-CBS1M, C7258∆p*tfoX*-CBS2M and C7258∆p*tfoX*-CBS1M+2M were significantly reduced compare to the wildtype strain. These results coincide with the above results of p*tfoX*^*VC*^-*lacZ*-CBS mutant fusions and indicate the regulation of TfoX-dependent natural transformation by CRP through the CBS sites *in vivo*.

CRP activates transcription by directly interacting with RNAP and/or acting upon DNA to change its structure to facilitate RNAP binding^34^. At the class I promoter, CRP binds upstream of the promoter and increases the rate of initial binding of RNAP to form a closed promoter complex (RPc). At the class II promoter, CRP binds within the promoter to increase the rate of transition from the closed to open promoter complex (RPo), in which the DNA duplex becomes unwound around the −10 region and short RNA products are synthesized^35^. We speculate that the loss of the CBS2 site might cause a substantial decrease in the −35 and −10 motif binding by RNAP in the *tfoX*^*VC*^ promoter and/or may decrease the efficiency of the transition from RPc to RPo. This will need to be investigated in the future studies. Another possibility is that an inverted repeat sequence (termed IR1), which was predicted to be a potential transcriptional operator^21^, overlaps the CBS2 site, so the site-directed mutagenesis of the consensus GTG to ACA in the CBS2 site could change the structure of IR1, thus affecting the transcription of *tfoX*^*VC*^. However, the present results demonstrate that the two putative CRP binding sites both play vital roles in the activation of *tfoX*^*VC*^expression.

### Determination of the TSS of *tfoX* ^*VF*^ and a comparative analysis of the *tfoX* promoter from different *Vibrio* species

The molecular structural features, such as the −10 and −35 motifs, transcriptional and translational *cis*-acting elements, secondary structure of the *tfoX*^*VC*^ mRNA, and TSS have already been determined^21^,^22^. The original annotated open reading frame (ORF) of *tfoX*^*VC*^was 609 bp long, with GUG as the start codon and GGGA as the predicted Shine-Dalgarno (SD) sequence. Through a series of mutational analyses of potential start codons based on fusion-plasmid constructions, Yamamoto revised the start codon and SD sequence of *tfoX*^*VC*^ to be ATG and GAAG, respectively, which are located 21 and 14 nucleotides downstream of the original sequences^21^,^22^. To assess the conservation of the molecular structural and regulatory characteristics of *tfoX* in different *Vibrio* species, we determined the TSS of *tfoX*^*VF*^ and compared the *tfoX* promoters in different *Vibrio* species whose genome sequences are available from the NCBI database and for which the homologs of *tfoX* have been annotated.

**Figure 5:**
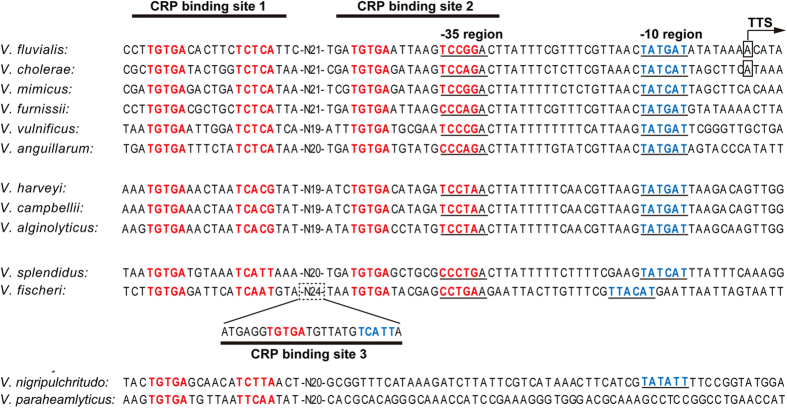
Structural organization of the *tfoX* promoter region. The *tfoX* promoter-proximate sequences from *V. fluvialis, V. cholerae, V. mimicus, V. furnissii, V. vulnificus, V. anguillarum, V. harveyi, V. campbellii, V. alginolyticus, V. splendidus, V. fischeri, V. nigripulchritudo* and *V. paraheamlyticus* were compared. The CRP binding sites are indicated with horizontal lines and the invert repeats in the CRP binding box are in boldface type. The putative −10 and/or −35 elements are underlined. The TTS of *V. fluvialis* and *V. cholerae* is designated with a box. The putative third CRP binding site located in the 24 bp spacing sequences in *V. fischeri* is also shown.

The open reading frame (ORF) of *tfoX*^*VF*^ shared 74.32% nucleotide identity with *tfoX*^*VC*^, but the intergenic regions showed much lower homology (55.87%). Using 5′ RACE, we identified a transcriptional start site 126 nucleotides upstream of the putative ATG start codon predicted according to the similarity to *tfoX*^*VC*^. Putative σ^70^- specific −10 (TATGAT) and −35 (TCCGGA) motifs separated by 19 bp were found in the upstream region of the TSS (Fig. 5), which coincides with the fact that competence genes are regulated by σ^70^ type promoters^36^. The −10 sequence has only one mismatch from the −10 motifs of *E. coli* (TATAAT) and *tfoX*^*VC*^(TATCAT). The −35 sequence has four mismatches from the *E. coli* consensus sequence TTGACA, but was a match at five of six nucleotides in the −35 region of *tfoX*^*VC*^(TCCAGA).

As shown in Fig. 5, conserved −10 motifs and two putative CRP-binding sites were found around similar locations in the *tfoX* promoter in *V. fluvialis*, *V. cholerae*, *V. mimicus*, *V. furnissii*, *V. vulnificus*, *V. anguillarum*, *V. harveyi*, *V. campbellii*, *V. alginolyticus*, and *V. splendidus*. The −35 regions were less conserved. This was not unexpected, because no CRP-dependent promoter has a good −35 sequence, and some even lack good −10 sequences^37^. The center-to-center distances between the two putative CRP binding sites were 43, 42 or 41 bp in different species. Three putative CRP-binding sites were discernible in the promoter region of *tfoX* in *V. fischeri*. Notably, the spacer sequence between the two half-sites of CRP binding site 3 in *V. fischeri* was seven bases, rather than the conventional six bases present in standard CRP half-sites. In general, the distal CRP-binding sites are more conserved and can roughly be classified into four classes based on the sequence homology, although the corresponding regions in *V. nigripulchritudo* and *V. paraheamlyticus* display higher variation. It is difficult to predict the putative −35 and/or −10 elements in similar locations in these species compared with the above species, and only one potential CRP binding site was found. The different numbers of binding sites and the variations in the binding sequences may contribute to the fine-tuning of *tfoX* expression in response to the changes in the environment, and may reflect dynamic evolutionary histories of the acquisition and or loss of CRP-binding sites.

### CRP binds directly to the *tfoX* promoter *in vitro*

**Figure 6:**
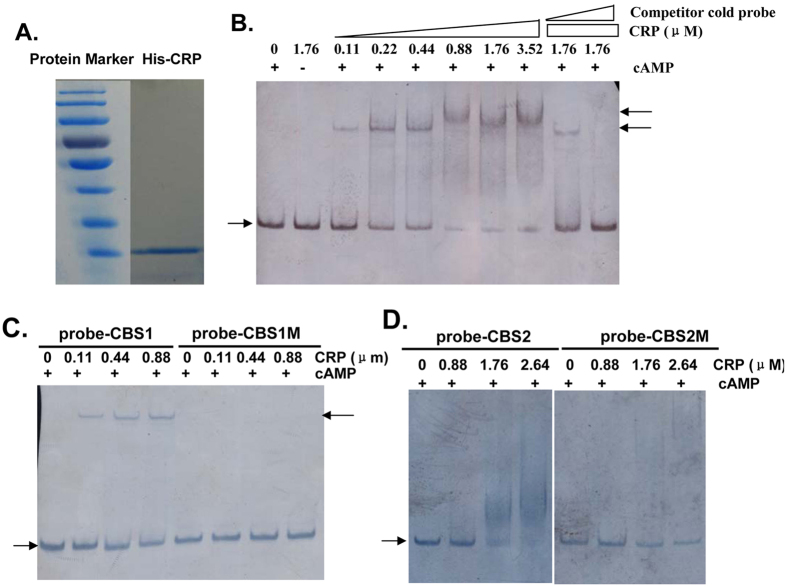
Binding of the cAMP-CRP complex to the promoter region of *tfoX*^*VC*^. EMSA were performed as described in the Methods section to determine whether there was a direct interaction between cAMP-CRP and the promoter region of *tfoX*^*VC*^. The arrow on the left side indicates the unbound free probe, whereas the arrow on the right side indicates the probe bound with CRP. (**A**) The purity of His-CRP analyzed with SDS-PAGE. (**B**) A biotin-labeled 152-bp DNA probe containing two CRP binding sites (10 ng) was incubated with increasing amounts of CRP in the presence of cAMP (0.1 mM). For the competition analysis, the same, but unlabeled, 152-bp DNA was included as 100-fold and 300-fold concentrations relative to the labeled probes. (**C**) The biotin-labeled 75-bp DNA fragments containing the original distal CRP binding site (probe-CBS1) or the corresponding mutagenized binding site (probe-CBS1M) were used as probes in a gel shift assay. (**D**) The biotin-labeled 97-bp DNA fragments containing the original proximal CRP binding site (probe-CBS2) or the corresponding mutagenized binding site (probe-CBS2M) were used as probes in the gel shift assay.

All of the above-described results suggest that *tfoX* expression is regulated at the transcriptional level via cAMP-CRP complex-mediated activation, and there are two cAMP-CRP binding sites at the *tfoX* promoter region. To verify whether there is cAMP-CRP binding to the putative sites, we measured the binding of purified CRP to a 152-bp DNA fragment of *tfoX*^*VC*^ encompassing the two CRP binding sites. The fragment was labeled with biotin at the 5′ end and used as a probe. As shown in Fig. 6B, the addition of CRP (0.11 μM) resulted in a shift of the 152-bp DNA fragment to slower mobility. When the amount of CRP was increased (>0.88 μM), the shifted band slowed to an even higher position. The binding was abolished when CRP or cAMP was excluded from the reaction mixture. Inclusion of the same, but unlabelled, 152-bp DNA fragment greatly competed with the labeled probe for the binding sites in a dose-dependent manner. The addition of 100-fold of the competitor cold probe changed the higher-shifted band to the lower one, while the addition of 300-fold of the cold probe completely abolished the retarded-band, and the labeled probe in the reaction mixture was released as the free probe. On one hand, these results confirmed the specific binding of CRP to the *tfoX*^*VC*^promoter, and on the other hand, suggested that one binding site exhibits lower-affinity binding. This is in agreement with the fact that the proximal CBS2 differs from the consensus sequence in three conserved positions, while CBS1 differs from the consensus sequence in only one conserved position, thus resulting in different binding affinity.

For further confirmation, we performed EMSA with the fragments containing single CRP binding sites. Consistent with the more conserved features of CBS1 and the EMSA results described above, the 75 bp fragment with CBS1 was shifted to a single band with slower mobility following the addition of 0.11 μM of CRP, while mutagenesis of the conserved GTG completely abolished the binding (Fig. 6C). While the 97 bp fragment with CBS2 was not shifted even when up to 0.88 μM of CRP was added, smeared bands were formed when more than 1.76 μM CRP were added in the assay mixture, which may indicate the instability of the DNA-CRP complex formed *in vitro* due to lower affinity of binding (Fig. 6D). Although the EMSA assays indicated that CBS2 is a low affinity binding site, the site-directed mutagenesis analysis demonstrated that this binding site also plays a vital role in the initiation of the transcription of *tfoX*^*VC*^. It should also be kept in mind that it is impossible to rule out the possibility that the low affinity binding of CBS2 was due to the *in vitro* binding conditions in the EMSA assay, which do not exactly mimic the *in vivo* condition.

With *V. cholerae* CRP, which has a high amino acid identity (99.05%) to the CRP in *V. fluvialis*, we also found the direct and specific binding of CRP to the *tfoX*^*VF*^ promoter in an EMSA assay (data not shown), further demonstrating the conserved direct regulatory effects of the cAMP-CRP complex on the competence regulator *tfoX* in *Vibrio* species.

## Discussion

Competence development for DNA uptake by microorganisms is tightly regulated, and cAMP-CRP plays a central role in competence regulation in response to nutritional stress. The direct binding of CRP to the CRE site on the promoter region of competence-associated genes was predicted^8^ and experimentally confirmed^10^. The second essential regulator of competence, TfoX, was proposed to act cooperatively with CRP by directing it to CRE sites to allow maximal expression of the competence genes^10^, although it lacks the helix-turn-helix DNA binding motif^12^ and remains recalcitrant to overexpression and purification^8^. Recently Jaskolska and coworker demonstrated that TfoX is unstable and degraded by Lon protease in *E.coli*^38^. Previously, Zulty & Barcak^39^ and Cameron *et al.*^20^ showed a strong induction of *sxy* expression after the addition of 1 mM cAMP to *H. influenzae,* although evidence for direct binding is not yet available. Additionally, the opposite result was also reported, i.e., *cya* mutation has no effect on *sxy* expression^10^. While we were in the preparation of the manuscript, Jaskolska *et al.* published their data showing that in *E. coli*, *Sxy* is positively autoregulated at the transcriptional level by a mechanism requiring cAMP-CRP and the CRP-S site in the *sxy* promoter^38^. It is interesting that *Vibrio* species possess the CRP-N sites instead of CRP-S sites in the *tfoX* promoter regions. Experimental evidences have demonstrated that the CRP-N sites are higher affinity sites than the CRP-S sites^10^,^12^,^36^. The presence of CRP-N sites in *tfoX* could start higher expression of TfoX and in turn more efficiently induce the development of competence, thus to favor the survival of *Vibrio* species in the aquatic system which is normally nutrition-limited. This finding further indicates that though the members of gamma-proteobacteria families, such as *Enterobacteriaceae, Pseudomonadaceae* and *Vibrionaceae* share a common regulatory mechanism in competence development^10^,^12^, the divergence or variation in details exists which may facilitate to refine the regulation in different genetic backgrounds and or survival environments. We reasoned that this may due to the long-term evolution pressure selection between the different native habitant environments.

In *V. cholerae*, chitin induces natural competence^5^, and cAMP and CRP are required for efficient chitin colonization, degradation and increased competence gene expression^19^. The regulation of *tfoX* by CRP, particularly in *Vibrio* species, had not been established or investigated. In the present study, we experimentally demonstrated that cAMP and CRP positively regulate the expression of *tfoX* in *V. cholerae* and *V. fluvialis*. A set of promoter deletion fusions and site-directed mutagenesis analyses confirmed the functional existence of two CRP binding sites in the *tfoX*^*VC*^ promoter region, and direct binding was further demonstrated by an EMSA *in vitro*.

A sequence comparison of the *tfoX* promoter region revealed the existence of optimal and suboptimal CRP-N binding sites in other *Vibrio* species homologs, suggesting that the transcriptional regulation of *tfoX* by CRP is a common feature in *Vibrionaceae*. The ecologies and natural hosts of *Vibrio* species vary, but free-living in sea water and attachment to zooplanktons are shared life stages. In general, sea water is a nutritionally-limited environment, where chitin is the most abundant nutrition alternative. The chitin utilization pathway has been known to be conserved in the *Vibrionaceae*^40^. Nutritional stress induces elevation of cAMP-CRP, and the availability of chitin efficiently activates *tfoX* expression. TfoX, as the early activated competence regulator, synergistically directs cAMP-CRP to the CRP-S sites on the competence regulon to further promote the competence development, thus allowing the bacteria to take up free DNA from the environment. The uptaken DNA can be used as either an energy source or to repair damaged DNA, or may be used to acquire new alleles/genes, which accounts for the intensive genetic diversity and the mosaic genome structure in *Vibrio* species revealed by recent genomic sequencing efforts^41^. It was thought that the reuse of nucleotides from DNA degradation in the cytoplasm may be more significant than other genetic benefits, at least in the short term^6^.

We also noticed that the degree of CRP binding sequence conservation varies and can roughly be classified into four different groups (Fig. 5). Generally, the distal CBS1 site is more conserved, with a match of at least eight of 10 nucleotides with the consensus binding site. In constrast, the half-site of the proximal CBS2 site obviously displays more sequence diversity, which may result in transient CRP-DNA interactions. It is tempting and logical to speculate that the regulatory features (such as the intensity and duration of binding) are distinct due to the differential binding affinity of CRP caused by sequence variations in different *Vibrio* species and between different binding sites. For example, *V. fischeri* possesses three regions that approximate the CRP consensus sequence. Even though *V. fischeri* has three CRP binding sites, the transformation efficiency has been reported to be 100-fold lower compared to that of *V. cholerae*^13^, indicating that the physiological significance of more binding sites needs to be established. The length of the spacer between the two CRP binding half-sites is usually 6 bp or 8 bp^42^,^43^. As mentioned before, the spacer region between the half-sites of the third CRP binding site in *V. fischeri* is 7 bp, which could potentially lead to a dramatic reduction in the binding affinity. The study by Pyles and Lee demonstrated that changing the spacer length from 7 bp to 8 bp increased the binding affinity of CRP for DNA^44^. Of course, considering the complexity of the competence-associated machinery components and their regulation, it cannot be stated this is the only reason for the 100-fold lower transformation.

In conclusion, our study provided a definitive analysis of the role of the cAMP-CRP in *tfoX* expression by experimentally demonstrating that the cAMP-CRP complex directly activates its transcriptional expression. These results, together with previous data^5^,^19^, demonstrate that the natural competence of *Vibrios* is subject to catabolite repression by the global transcriptional regulator, CRP, and this regulation is effected through direct control of both the vital competence regulator gene and the competence component genes. In addition, CRP indirectly regulates competence through quorum-sensing by activating primary autoinducer synthesis gene, *cqsA*^27^,^28^. The multiple forms of regulation at different layers or pathways mediated by CRP may maximize the input of simple environmental signals to induce or promote the development of competence for the efficient uptake of extracellular DNA as a nutritional supply or for other purposes, thus greatly favoring the survival and environmental fitness of the organism.

## Methods

### Strains and media

The *V. cholerae* and *V. fluvialis* mutants used in this study were constructed using the El Tor biotype strain, C7258 (Peru′ isolate, 1991), and the clinical strain, 85003 45, as wild-type precursors, respectively. The construction of strains WL7258 (C7258∆crp), WL7259 (C7258∆cya), C7258∆lacZ and WL7258∆lacZ has been described previously^27^,^28^,^46^. pBADCRP7 was introduced into WL7258∆lacZ by electroporation^47^. *Escherichia coli* DH5α*λpir* and S17-1*λpir* were used for cloning purposes. The *V. cholerae* and *V. fluvialis* strains were grown in LB broth containing 1% NaCl at 37 °C with agitation (250 rpm). Culture media were supplemented with ampicillin (Amp, 100 μg/ml), kanamycin (Km, 75 μg/ml), chloramphenicol (Cm, 5 μg/ml), streptomycin (Sm, 100 μg/ml), or polymyxin B (PolB, 100 units/ml) as required. All strains, plasmids and primers used in this study are listed in Table 1.

### Construction of mutants

The *V. fluvialis* deletion mutant was constructed from clinical strain 85003 by allelic exchange^45^. Primers were designed based on the genome sequence of the *V. fluvialis* 85003 deposited in the Sequence Read Archive under accession no. SRX397301 45. To construct the ∆*crp* mutant, 85003∆*crp*, the upstream and downstream DNA fragments flanking the *crp* open reading frame (ORF) were amplified from 85003 genomic DNA using primer pairs VF-CRP-F1-up-*Sal*I /VF-CRP-F1-dn and VF-CRP-F2-up/VF-CRP-F2-dn-*Sac*I, respectively. The amplicons were mixed in equimolar concentration and used as the template to amplify the chromosomal fragment containing the *crp* deletion using primer pair VF-CRP-F1-up-*Sal*I/VF-CRP-F2-dn-*Sac*I. The resulting fragment was cloned into suicide plasmid pWM91 to generate pWM-VF∆*crp*. pWM-VF∆*crp* was constructed in *E. coli* S17-1*λpir* and transferred to strain 85003 by conjugation. Exconjugants were selected in LB medium containing Amp and Sm, and were streaked on LB agar containing 15% (w/v) sucrose. Sucrose-resistant colonies were tested for Amp sensitivity, and the deletion of *crp* was confirmed by DNA sequencing.

### Construction of transcriptional reporter plasmids

To construct the p*tfoX*^*VC*^–*lacZ* transcriptional fusion, we cloned a 430-bp fragment containing the *tfoX*^*VC*^ promoter region amplified with primers pVC1153-up-*Pst*I/pVC1153-dn-*Hind*III into pTT3 48 downstream of the strong *rrnB*T1T2 transcription terminator to generate pTT-*tfoX*^*VC*^. Next, a 900 bp *Kpn*I-*Hind*III fragment containing the *rrnB*T_1_T_2_ terminator and the *tfoX*^*VC*^ promoter from pTT-*tfoX*^*VC*^ was ligated into the big *Hind*III-*Kpn*I fragment of phaplac7, which contains a promoterless *lacZ* gene, the pBR322 origin of replication, and the *bla* gene^48^. The resultant plasmid, p*tfoX*^*VC*^-*lacZ*, was introduced into C7258∆lacZ and WL7258∆lacZ by electroporation^47^. Additional DNA fragments harboring 5′ deletions of the *tfoX*^*VC*^ promoter were amplified with primer pairs of pVC1153-up2-*Pst*I/pVC1153-dn-*Hind*III and pVC1153-up3-*Pst*I /pVC1153-dn-*Hind*III, and transcriptional fusions p2TT*tfoX*^*VC*^ and p3TT*tfoX*^*VC*^ were constructed in a similar manner. To construct the bioluminescence based p*tfox*^*VF*^-*lux* fusion, we cloned a 384 bp fragment containing the *tfoX*^*VF*^ promoter region amplified with primers VF-sxy-prom-up-*Sac*I/VF-sxy-prom-dn-*BamH*I into reporter plasmid pBBR*lux*, which contains a promoterless *luxCDABE* operon^49^. p*tfox*^*VF*^-*lux* was constructed in DH5α*λpir* and transferred into S17-1*λpir*, and then mobilized into *V. fluvialis* strains 85003 and 85003∆*crp* by conjugation.

### Quantitative reverse transcription PCR (qRT-PCR)

The *V. cholerae* and *V. fluvialis* strains were grown in LB medium to the late-log phase. Cells were collected by centrifugation at 4 °C and immediately subjected to RNA extraction. Total RNA was extracted using the Trizol reagent (Ambion), followed by treatment with a TURBO DNA-free^TM^ kit (Ambion) to remove chromosomal DNA contamination. The purity and integrity of RNA samples was verified by UV spectrophotometry and agarose gel electrophoresis. Using 1 μg of total RNA per sample, cDNA was synthesized using random hexamer primers with SuperScript^TM^ III reverse transcriptase (Invitrogen) according to the manufacturer’s instructions. qRT-PCR was performed using SYBR Green (TaKaRa) on the Bio-Rad CFX96 Real-Time PCR detection system. The relative expression values (R) using *recA* mRNA as a reference were calculated using the equation R = 2^−(ΔCq target −ΔCq reference)^, where Cq is the fractional threshold cycle. The following primer combinations were used: VC1153-up and VC1153-dn for *tfox*^*VC*^; recA578 and recA863 for *recA*^*VC *^27; VF-sxy-qPCR-up and VF-sxy-qPCR-dn for *tfox*^*VF*^; VF-recA-qPCR-up and VF-recA-qPCR-dn for *recA*^*VF*^; *pilB*-real-u and *pilB*-real-d for *pilB*; *chiA*1-real-u and *chiA*1-real-d for *chiA*-1; *chiA*2-real-u and *chiA*2-real-d for *chiA*-2. A control mixture with total RNA as a template was used in each reaction to exclude the effects of chromosomal DNA contamination.

### Determination of the 5′-end of *tfoX* ^*VF*^ mRNA

We used 5′ RACE (rapid amplification of cDNA ends) to determine the TSS of *tfoX*^*VF*^. *V. fluvialis* strain 85003 was grown in LB containing 1% NaCl at 37 °C with agitation to OD600 1.5. Total RNA was extracted as described in the preceding section. The cDNA was generated using the SMARTer™ RACE cDNA Amplification Kit (Clontech) according to the manufacturer’s instructions. The cDNA was then amplified by PCR using a kit provided with UPM (universal primer) and the gene specific primer, VF-sxy-race. The PCR product obtained was gel-purified and cloned into the pMD^®^18-T vector. Ten clones were sequenced using the primers M13-R and M13-F to determine the TSS.

### Site-directed mutagenesis of the putative CRP binding sites of the *tfoX* ^*VC*^ promoter

The two putative CRP binding sites in the promoter region of *tfoX*^*VC*^were mutated using the Hieff Mut^TM^ site-directed mutagenesis kit (Shanghai YEASEN Biotechnology Co., Ltd.) according to the manufacturer’s instructions. The above constructed p*tfoX*^*VC*^-*lacZ* plasmid containing the full-length functional *tfoX* promoter region was used as the template, and mutagenesis was induced using primers carrying the substituted nucleotides. Primer pairs VC1153CBS1M-for/VC1153CBS1M-rev and VC1153CBS2M-for/VC1153CBS2M-rev were used to substitute three bases in the half-site of putative CRP binding sites 1 and 2, respectively. To simultaneously mutate the two CRP binding sites, primer combinations VC1153CBS1M-for/VC1153CBS2M-rev and VC1153CBS1M-rev/ VC1153CBS2M-for were used. The resultant plasmids, p*tfoX*^*VC*^-*lacZ*-CBS1M, p*tfoX*^*VC*^-*lacZ*-CBS2M and p*tfoX*^*VC*^-*lacZ*-CBS1+2M, were sequenced to confirm the mutations. Subsequently, the obtained plasmid was introduced into *V. cholerae* strain C7258∆*lacZ* by electroporation.

Mutations in CBS1M, CBS2M and CBS1M+2M were further introduced into the chromosomal promoter region of *tfoX* of C7258 through suicide plasmid-mediated allelic exchange. For this purpose, the promoter region of *tfoX* was first replaced with a kanamycin gene, yielding strain C7258∆p*tfoX*-*kan*. Then, C7258∆*ptfoX*-*kan* was used as precursor to introduce CBS1M, CBS2M and CBS1M+2M mutations on the chromosome. To construct C7258∆p*tfoX*-*kan*, the flanking sequence of the *tfoX* promoter region was amplified with primer pairs F1-up-*Sal*I/ F1-dn-*Pst*I and F3-up-*Pst*I/ F3-dn-*Sph*I, and was sequentially cloned into pCVD442, then the *kan* gene was inserted at the *Pst*I site. The resulting plasmid, pCVD-∆p*tfoX*-*kan*, was mobilized into C7258 by conjugation, and sucrose selection was applied to isolate segregants containing the kan replacement of the *tfoX* promoter region.

Subsequently, three recombinant suicide plasmids, pCVD-∆p*tfoX*-CBS1M, pCVD-∆p*tfoX*-CBS2M and pCVD-∆p*tfoX*-CBS1M+2M were generated. Primer pairs F1-up-*Sal*I/F1-dn and F3-up/F3-dn-*Sph*I were used to amplify the flanking sequence. Plasmids p*tfoX*^*VC*^-*lacZ*-CBS1M, p*tfoX*^*VC*^-*lacZ*-CBS2M and p*tfoX*^*VC*^-*lacZ*-CBS1+2M were used as a template to amplify the *tfoX* promoter region containing the CBS1M, CBS2M and CBS1M+2M mutations with the primer pair F2-up/F2-dn. Bridge PCR was then applied to join the three fragments, which were further cloned as *Sal*I/*Sph*I fragments into pCVD442. The resulting plasmids pCVD-∆p*tfoX*-CBS1M, pCVD-∆p*tfoX*-CBS2M and pCVD-∆p*tfoX*-CBS1M+2M were transferred into C7258∆p*tfoX*-*kan*. Exconjugants were selected in LB medium containing Amp and PolB and sucrose selection was applied to isolate segregants retaining the CBS1M, CBS2M and CBS1M+2M alleles. The corresponding mutants were named C7258∆p*tfoX*-CBS1M, C7258∆p*tfoX*-CBS2M and C7258∆p*tfoX*-CBS1M+2M, respectively.

### Expression and purification of His-CRP

*E. coli* TOP10 harboring the recombinant plasmid pBADCRP7^50^ were cultured at 37 °C in LB medium. The 6xHis-tagged CRP was induced by 0.2% (w/v) arabinose and purified by Ni-IDA affinity chromatography (Novagen) under native conditions according to the manufacturer’s instructions. The purity was analyzed by SDS-PAGE (Fig. 6A), and the concentration was determined with a Pierce BCA Protein Assay kit.

### Electrophoretic mobility shift assays (EMSA)

EMSA was performed as described previously^51^. The 152 bp, 75 bp and 97 bp fragments of the *tfoX*^*VC*^ promoter regions were amplified with the biotin-labeled primer pairs Shift-up1976-96/Shift-dn2107-27, Shift-up1976-96/VC1153-p1shift-dn, and VC1153-p2shift-up/Shift-dn2107-27 respectively, and were used as probes. The 152 bp probe covers fragments ranging from −127 to 24 relative to the reported TSS^21^ and encompasses two putative CRP binding sites. The same fragments without a biotin label were used as competing cold probes and were added in 100–300-fold excess of the labeled probes. The 75 bp and 97 bp probes extend from residues −127 to −53 and −74 to 24, respectively, and each contains a single CRP binding site. Binding reactions were performed by mixing each biotin-labeled probe with increasing quantities of purified CRP in 10 μl of reaction volume with binding buffer (50 mM Tris-HCl [pH7.8], 250 mM KCl, 5 mM MgCl_2_, 2.5 mM EDTA, 0.5 mM MnCl_2_, 2.5 mM DTT, 1 μg Poly (dI.dC) and 100 μM cAMP). The reaction mixture was incubated at room temperature for 30 min, and then separated on a 6% (for 152 bp probe) or 10% (for 75 bp and 97 bp probes) native polyacrylamide gel after adding loading buffer. The separated DNA and DNA-protein samples were transferred onto nylon membranes using the Mini Trans-Blot Electrophoretic Transfer cell (Bio-Rad), and were detected with the Chemiluminescent Nucleic Acid Detection Module (Thermo Scientific) following the manufacturer’s instructions.

### *β*-Galactosidase assays and bioluminescence assays

Overnight cultures of *V. cholerae* strains C7258∆lacZ and WL7258∆lacZ containing *tfoX*^*VC*^-*lacZ* fusion plasmids were diluted 100-fold into fresh LB, and were grown to mid-exponential phase. The specific activities of *β*-galactosidase are expressed in Miller units [1000 (OD420/*t v* OD600)], where *t* is the reaction time and *v* is the volume of enzyme extract per reaction^52^. Similarly, *V. fluvialis* strains 85003 and VF∆*crp* with p*tfox*^*VF*^-*lux* were cultured to the mid-exponential phase. The absorbance and bioluminescence were quantified thereafter. The bioluminescence was measured on opaque-wall 96-well microtiter plates (ostar 3917) with a Tecan Infinite M200 Pro luminometer. The promoter activity is expressed as Lux/OD600.

## Additional Information

**How to cite this article**: Wu, R. *et al.* Direct regulation of the natural competence regulator gene *tfoX* by cyclic AMP (cAMP) and cAMP receptor protein (CRP) in *Vibrios*. *Sci. Rep.* **5**, 14921; doi: 10.1038/srep14921 (2015).

## Figures and Tables

**Table 1:** Strains, plasmids and primers used in this study.

Strain, plasmid or primer	Characteristics or sequence
Strains	
C7258	*V. cholerae*, wild type, El Tor biotype (Peru´ isolate,1991)
WL7258	C7258, ∆*crp*^27^
WL7259	C7258, ∆*cya*^28^
C7258∆lacZ	C7258, ∆*lacZ*^46^
WL7258∆lacZ	C7258, ∆*crp*, ∆*lacZ*^46^
S17-1*λpir*	*thi thr leu tonA lacY supE recA*: :RP4-2Tc : :Mu (*λpir*R6K) (Laboratory stock)
85003	*V. fluvialis*, wild type, Sm^R ^45
85003∆*crp*	85003, ∆*crp* (This study)
C7258∆p*tfoX*-CBS1M	C7258 containing the site-specific mutagenesis of CRP binding site 1 on the chromosomal *tfoX*^*VC*^ promoter region (This study)
C7258∆p*tfoX*-CBS2M	C7258 containing the site-specific mutagenesis of CRP binding site 2 on the chromosomal *tfoX*^*VC*^ promoter region (This study)
C7258∆p*tfoX*-CBS1M+2M	C7258 containing the site-specific mutagenesis of CRP binding site 1 and 2 on the chromosomal *tfoX*^*VC*^ promoter region (This study)
Plasmids	
pWM91	Suicide vector containing R6K *ori*, *sacB*, *lacZα*; Amp^R^ (Laboratory stock)
pCVD442	Suicide vector containing R6K *ori*, *sacB*, Amp^R^ (Laboratory stock)
pWM-VF∆*crp*	1 kb *Sal*I-*Sac*I ∆*crp* fragment of *V. fluvialis* in pWM91 (This study)
pTT3	*rrnB*T1T2 transcription terminator in pUC19; Amp^R ^48
pTT-*tfoX*^*VC*^	430 bp *Pst*I- *Hind*III fragment of *tfoX*^*VC*^ promoter region in pTT3 (This study)
phaplac7	hapR promoter cloned in pKRZ1 in front of promoterless *lacZ* gene; Amp^R ^48
p*tfoX*^*VC*^-*lacZ*	*Hind*III-*Kpn*I fragment containing *rrnB*T1T2 and *tfoX*^*VC*^ promoter from pTT-*tfoX*^*VC*^ in phaplac7 (This study)
p2TT*tfoX*^*VC*^	220 bp *Pst*I- *Hind*III fragment of *tfoX*^*VC*^ promoter region in p*tfoX*^*VC*^-*lacZ* (This study)
P3TT*tfoX*^*VC*^	185 bp *Pst*I- *Hind*III fragment of *tfoX*^*VC*^ promoter region in p*tfoX*^*VC*^-*lacZ* (This study)
pBBR*lux*	bioluminescence based reporter plasmid containing a promoterless *luxCDABE* operon; Cm^R ^49
p*tfox*^*VF*^-*lux*	384 bp *Sac*I-*BamH*I fragment of *tfoX*^*VF*^promoter region in pBBR*lux* (This study)
p*tfoX*^*VC*^-*lacZ*-CBS1M	p*tfoX*^*VC*^-*lacZ* with the mutagenized sequence for the putative CRP binding site 1 (This study)
p*tfoX*^*VC*^-*lacZ*-CBS2M	p*tfoX*^*VC*^-*lacZ* with the mutagenized sequence for the putative CRP binding site 2 (This study)
p*tfoX*^*VC*^-*lacZ*-CBS1+2M	p*tfoX*^*VC*^-*lacZ* with the mutagenized sequence for the putative CRP binding site 1 and 2 (This study)
pBADCRP7	*V. cholerae crp* ORF cloned in pBADHisB^50^
pCVD-∆p*tfoX*-*kan*	3.0 kb *Sal*I-*Sph*I fragment containing the flanking sequence of *tfoX*^*VC*^promoter region and kan gene in pCVD442 (This study)
pCVD-∆p*tfoX*-CBS1M	2.158 kb *Sal*I-*Sph*I fragment containing the *tfoX*^*VC*^ promoter region with the mutagenized sequence for the putative CRP binding site 1 and the flanking sequence in pCVD442 (This study)
pCVD-∆p*tfoX*-CBS2M	2.158 kb *Sal*I-*Sph*I fragment containing the *tfoX*^*VC*^ promoter region with the mutagenized sequence for the putative CRP binding site 2 and the flanking sequence in pCVD442 (This study)
pCVD-∆p*tfoX*-CBS1M+2M	2.158 kb *Sal*I-*Sph*I fragment containing the *tfoX*^*VC*^ promoter region with the mutagenized sequence for both the putative CRP binding sites and the flanking sequence in pCVD442 (This study)
Primers	
VF-CRP-F1-up-*Sal*I	5′-AACGTCGACTACCCTTACCTGC-3′
VF-CRP-F1-dn	5′-GTGACGATTACAAAGTCTCTGCTTTTTCG-3′
VF-CRP-F2-up	5′-AGAGACTTTGTAATCGTCACCGAGACAGAA-3′
VF-CRP-F2-dn-*Sac*I	5′-CGAGCTCCGTCTGTGGGATCTGAG-3′
pVC1153-up-*Pst*I	5′-ACCTGCAGCGGGTAACCAGTAAAAAG-3′
pVC1153-dn-*Hind*III	5′-CCCAAGCTTCGAAAAACTGTTGCTCAT-3′
VF-sxy-prom-up-*Sac*I	5′-CGAGCTCCATTGTTTATCATTGTTAGT-3′
VF-sxy-prom-dn-*BamH*I	5′-CGGGATCCCATATCCATTGATCCTTTAA-3′
VF-sxy-qPCR-up	5′-CGTTCTATGTTTGGTGGTATTG-3′
VF-sxy-qPCR-dn	5′-GCCGTTGTCTGCTTCTTC-3′
VF-recA-qPCR-up	5′-ACCGAGTCAACGACGATAAC-3′
VF-recA-qPCR-dn	5′-TGATGAACTGCTGGTGTCTC-3′
VC1153-up	5′-ACGCTCGATGTTTGGTGGTATTGG-3′
VC1153-dn	5′-ATTGGGTAAATCCCGTAGACGACG-3′
VF-sxy-race	5′-TCTGCTTCTTCACATGACGG-3′
Shift-up1976/1996	5′-GGTTTAAGTATAGAGGAGCA-3′(5′ biotin-labeled)
Shift-dn2107/2127	5′-GTTCGTATGAGCTTGCTTGT-3′(5′ biotin-labeled)
VC1153-p1shift-dn	5′-ATGCAATACTTTTGCGCCAG-3′(5′ biotin-labeled)
VC1153-p2shift-up	5′-CTGGCGCAAAAGTATTGCAT-3′(5′ biotin-labeled)
VC1153CBS1M-for	5′-TTGACGCTCACATACTGGTCTCATAATCTGG-3′
VC1153CBS1M-rev	5′-GACCAGTATGTGAGCGTCAATTTTTTGTTGC-3′
VC1153CBS2M-for	5′-TGCATCGATCACAGATAAGTCCAGACTTATTTCTC-3′
VC1153CBS2M-rev	5′-GACTTATCTGTGATCGATGCAATACTTTTGCGCCA-3′
F1-up-*Sal*I:	5′-GCGTCGACATCACTTAGCTTGTTGTT-3′
F1-dn-*Pst*I:	5′-GCTCATCTGCAGCTTTTTACTGGTTACCCG-3′
F2-up:	5′-GTAACCAGTAAAAAGATAGGTGACTCATAA-3′
F2-dn:	5′-ACTGTTGCTCATTCATATCCATTGATCCTT-3′
F3-up-*Pst*I:	5′-AAAAGCTGCAGATGAGCAACAGTTTTTCGA-3′
F3-dn-*Sph*I:	5′-CATGCATGCATTGGTATTCGTCAGTGG-3′
*pilB*-real-u	5′-ATGCTCACCAACCTTGTT-3′
*pilB*-real-d	5′-TGTAACCACCGCTTGTTC-3′
*chiA*1-real-u	5′-TGGCATAACTTCGTCAAT-3′
*chiA*1-real-d	5′-AAGGCAATATCAATCACATC-3′
*chiA*2-real-u	5′-GCTATCGGTGTTGGTCAT-3′
*chiA*2-real-d	5′-CGTAGAAGTCATAAGTCATTGC-3′
